# Management of Human Breast-Milk in Cases of Difficult Infantile Digestion

**Published:** 1890-08

**Authors:** 


### MANAGEMENT OF HUMAN BREAST-MILK IN CASES OF DIFFICULT INFANTILE DIGESTION.

At the meeting of the American Pediatric Society, on June 3, 1890, Dr. T. M. Rotch, of Boston, in this paper gave an analysis of the human milk in a series of cases, during the time of healthy digestion, and also during indigestion in the infant, showing at the same time the bearing of the mother's health and habits, and the amount and quality of food taken, upon the character of the milk (*Medical Record*, June 14, 1890).

Physicians acknowledged that the maternal breast-milk (meaning thereby the average) was the best diet for the infant, but did not seem generally to fully realize the importance of their admission, for not infrequently they forbade the use of this food, and ordered a wet-nurse or hand-feeding, when the infant showed indisposition more or less marked, without taking the trouble to analyze the milk, or inquire into the reasons for its possible unwholesome qualities. Brief reference was made to the various conditions affecting the quantity and quality of the milk, such as the state of the mother's health, exercise, food, mental emotions, length of the intervals between the nursings, etc. The longer the intervals the greater the proportion of the watery elements. In a general way the milk could be looked upon in its normal condition as a secretion, yet as colostrum and under certain unfavorable conditions it took on to a considerable degree the character of an excretion. Marked variations in the milk were almost always shown in the amount of fat and albuminoids which it contained; and, consequently, we treated almost universally these elements. Meat and a nitrogenous diet increased the fats in milk, while a diet of fat had not this effect. The albuminoids were more difficult to deal with. To lessen them, he had found in practice the best means was, if the woman were in health, to increase the amount of exercise, preferably walking in the open air. One or two miles a day was the walk required by the average woman. In the diagrams presented, containing an analysis of the milk in several cases of difficult digestion, there was usually an increase in the amount of albuminoids, sometimes up to or beyond four per cent., or with or without a change in the fats, and patient had the effect of reducing the albuminoid. He had not known an abscess to follow the use of the pump on the human breast, and exhibited a simple and efficient instrument of this kind. If one had to deal with a case in which there was very poor milk, this should first be made rich, and afterwards the constituents be altered as required. But there were cases in which, in spite of following out the rules contained in the paper, we were unable to manage the milk, and make it suitable food for the infant. Nor were there fixed limits as to the proportion of albuminoids and other ingredients that should be present. Each case must be studied by itself.

